# Berberine Inhibits the Release of Glutamate in Nerve Terminals from Rat Cerebral Cortex

**DOI:** 10.1371/journal.pone.0067215

**Published:** 2013-06-19

**Authors:** Tzu-Yu Lin, Yu-Wan Lin, Cheng-Wei Lu, Shu-Kuei Huang, Su-Jane Wang

**Affiliations:** 1 Department of Anesthesiology, Far-Eastern Memorial Hospital, New Taipei, Taiwan; 2 Graduate Institute of Basic Medicine, Fu Jen Catholic University, New Taipei, Taiwan; 3 Department of Mechanical Engineering, Yuan Ze University, New Taipei, Taiwan; Aston University, United Kingdom

## Abstract

Berberine, an isoquinoline plant alkaloid, protects neurons against neurotoxicity. An excessive release of glutamate is considered to be one of the molecular mechanisms of neuronal damage in several neurological diseases. In this study, we investigated whether berberine could affect endogenous glutamate release in nerve terminals of rat cerebral cortex (synaptosomes) and explored the possible mechanism. Berberine inhibited the release of glutamate evoked by the K^+^ channel blocker 4-aminopyridine (4-AP), and this phenomenon was prevented by the chelating extracellular Ca^2+^ ions and the vesicular transporter inhibitor bafilomycin A1, but was insensitive to the glutamate transporter inhibitor DL-threo-beta-benzyl-oxyaspartate. Inhibition of glutamate release by berberine was not due to it decreasing synaptosomal excitability, because berberine did not alter 4-AP-mediated depolarization. The inhibitory effect of berberine on glutamate release was associated with a reduction in the depolarization-induced increase in cytosolic free Ca^2+^ concentration. Involvement of the Ca_v_2.1 (P/Q-type) channels in the berberine action was confirmed by blockade of the berberine-mediated inhibition of glutamate release by the Ca_v_2.1 (P/Q-type) channel blocker ω-agatoxin IVA. In addition, the inhibitory effect of berberine on evoked glutamate release was prevented by the mitogen-activated/extracellular signal-regulated kinase kinase (MEK) inhibitors. Berberine decreased the 4-AP-induced phosphorylation of extracellular signal-regulated kinase 1 and 2 (ERK1/2) and synapsin I, the main presynaptic target of ERK; this decrease was also blocked by the MEK inhibition. Moreover, the inhibitory effect of berberine on evoked glutamate release was prevented in nerve terminals from mice lacking synapsin I. Together, these results indicated that berberine inhibits glutamate release from rats cortical synaptosomes, through the suppression of presynaptic Cav2.1 channels and ERK/synapsin I signaling cascade. This finding may provide further understanding of the mode of berberine action in the brain and highlights the therapeutic potential of this compound in the treatment of a wide range of neurological disorders.

## Introduction

Berberine is an isoquinoline alkaloid and present in many medicinal herbs, such as *Berberis*, *Hydrastis canadensis* and *Coptidis rhizoma* [1]. Berberine has multiple pharmacological activities including anti-inflammatory, antitumor, antimalarial, antioxidative and cardioprotective effects [2]. In addition to these properties, berberine has numerous beneficial effects in the central nervous system (CNS), in particular, neuroprotective effect [3]. For example, berberine protects against ischemia- or β-amyloid-induced neuronal death [4], [5], [6], [7], and ameliorates β-amyloid-induced memory impairment [8], [9]. Based on these reports, berberine might be a potential candidate for a natural neuroprotective agent. The mechanisms underlying the neuroprotective effects of berberine are, however, not fully clarified.

In the mammalian central nervous system (CNS), glutamate is the major excitatory neurotransmitter and plays an important role in many functions such as cognition, movement, learning, and memory [10]. Besides its physiological role, excessive glutamate release and activation of glutamate receptors induces an increase in intracellular Ca^2+^ levels, which in turn triggers a cascade of cellular responses, including enhanced oxygen free radical production, disturbed mitochondrial function, and protease activation, that ultimately kill the neurons [11]. This type of over-excitation-induced neuronal damage is not only involved in acute insults such as stroke, epileptic seizures, traumatic brain and spinal cord injury, but also in chronic neurodegenerative disorders such as Alzheimer's disease, Parkinson's disease, and amyotrophic lateral sclerosis [12], [13]. Thus, if a compound can attenuate glutamate release from nerve terminals, it may have a neuroprotective effect on the pathological conditions related to excessive glutamate release. Recently, some plant-derived compounds having neuroprotective property, such as hypericin (a active component of St. John's wort), curcumin (a active component of turmeric), fangchinoline (a active component of radix stephaniae tetrandrinea), and tanshinone IIA (a active component of danshen), have been demonstrated to decrease glutamate release from rat cortical nerve terminals [14], [15], [16], [17]. Berberine has a neuroprotective-like effect and whether berberine has an effect on endogenous glutamate release should be evaluated.

The aim of the present study was to use isolated nerve terminals (synaptosomes) purified from the rat cerebral cortex to investigate the effect of berberine on the levels of endogenous released glutamate and to characterize its underlying molecular mechanisms. The isolated presynaptic terminal represents a model system for investigating directly the molecular mechanisms underlying presynaptic phenomena. Specifically, this preparation is capable of accumulating, storing, and releasing neurotransmitters, and is devoid of functional glial and nerve cell body elements that might obscure interpretation because of modulatory loci at non-neuronal, postsynaptic, or network levels [18]. The first series of experiments determined the effects of berberine on the release of endogenous glutamate, the synaptosomal plasma membrane potential, and the activation of voltage-dependent Ca^2+^ channels (VDCCs). In view of the demonstrated role of mitogen-activated protein kinase (MAPK) in presynaptic modulation [19], [20], the second series of experiments determined whether this signaling pathway participates in the regulation of berberine on glutamate release. Our results showed that berberine inhibits glutamate release from cortical synaptosomes in rats through the suppression of presynaptic voltage-dependent Ca^2+^ entry and ERK/synapsin I signaling pathway.

## Materials and Methods

### Chemicals

Bafilomycin A1, DL-*thre*o-β-benzyloxyaspartate (DL-TBOA), dantrolene, 7-chloro-5-(2-chlorophenyl)-1,5-dihydro-4,1-benzothiazepin-2(3*H*)-one (CGP37157), ω-conotoxin GVIA (ω-CgTX GVIA), ω-agatoxin IVA (ω-AgTX IVA) 2-(2-amino-3-methoxyphenyl)-4*H*-1-benzopyran-4-one (PD98059), and ***N***-(Cyclopropylmethoxy)-3,4,5-trifluoro-2-[(4-iodo-2-methylphenyl)amino]-benzamide (PD198306) were purchased from Tocris Cookson (Bristol, UK). Fura-2-acetoxy-methyl ester (Fura-2-AM), and 3′, 3′, 3′-dipropylthiadicarbocyanine iodide [DiSC_3_(5)] were bought from Invitrogen (Carlsbad, CA, USA). Rabbit polyclonal antibodies directed against ERK1/2 and phospho-ERK1/2 were bought from Cell Signaling Technology (Beverly, MA, USA). The anti-synapsin I phosphorylation state-specific rabbit polyclonal antibody directed against MAPK/ERK-phosphorylated sites 4, 5 of synapsin I (Ser^62^/Ser^67^) was purchased from Millipore (MA, USA). Horseradish peroxidase-conjugated anti-rabbit secondary antibodies were purchased from BioRad (Milan, Italy). Berberine, ethylene glycol bis (β-aminoethyl ether)-N,N,N′,N′-tetraacetic acid (EGTA), sodium dodecyl sulfate (SDS), and all other reagents were purchased from Sigma-Aldrich Co. (St. Louis, MO, USA).

### Animals

Adult male Sprague–Dawley rats (150–200 g) or six-week old male wild-type mice or synapsin I-deficient (SYN I−/−) mice were used in this study. Heterozygous synapsin I knockout (B6;129S-*Syn1^tm1Sud^*/J) female (+/−) and wild type male (+/y) mice were purchased from the Jackson Laboratory (Stock no. 002444, Bar Harbor, ME, USA). Synapsin I-deficient mice were generated as described previously [21]. All animal procedures were carried out in accordance with the National Institutes of Health Guidelines for the Care and Use of Laboratory Animals (NIH Publication No. 85–23, revised 1996), and were approved by the Fu Jen Institutional Animal Care and Utilization Committee. All efforts were made to minimize animal suffering and to reduce the number of animals used.

### Synaptosomal preparation

Synaptosomes were purified by discontinuous Percoll gradients as previously described [18]. Briefly, the animals were killed by decapitation and the cerebral cortex were rapidly removed at 4°C. The tissue was homogenized in a medium containing 320****mM sucrose, pH 7.4. The homogenate was centrifuged at 3000****g (5000****rpm in a JA 25.5 rotor; Beckman Coulter, Inc., USA) for 10****min at 4°C, and the supernatant was centrifuged again at 14,500****g (11 000****rpm in a JA 25.5 rotor) for 12****min at 4°C. The pellet was gently resuspended in 8****ml of 320****mM sucrose, pH 7.4. Two milliliters of this synaptosomal suspension was placed into 3****ml Percoll discontinuous gradients containing 320****mM sucrose, 1****mM EDTA, 0.25****mM DL-dithiothreitol, and 3, 10 and 23% Percoll, pH 7.4. The gradients were centrifuged at 32,500****g (16 500****rpm in a JA 20.5 rotor) for 7****min at 4°C. Synaptosomes sedimenting between the 10 and the 23% Percoll bands were collected and diluted in a final volume of 30****ml of HEPES buffer medium (HBM) consisting of 140****mM NaCl, 5****mM KCl, 5****mM NaHCO_3_, 1****mM MgCl_2_⋅6H_2_O, 1.2****mM Na_2_HPO_4_, 10****mM glucose, and 10****mM HEPES (pH 7.4). Protein concentration was determined using the Bradford assay. Synaptosomes were centrifuged in the final wash to obtain synaptosomal pellets with 0.5****mg protein. Synaptosomal pellets were stored on ice and used within 4–6****h.

### Glutamate release

Glutamate release was assayed by on-line fluorometry [22], [23]. Pelleted synaptosomes were resuspended at a protein concentration of 0.5****mg/ml in HBM containing 16 µM bovine serum albumin (BSA) and incubated in a stirred and thermostatted cuvette at 37 °C in a Perkin-Elmer LS-55 spectrofluorimeter (PerkinElmer Life and Analytical Sciences, Waltham, Mass., USA.). NADP^+^ (2 mM), glutamate dehydrogenase (50 units/ml) and CaCl_2_ (1.2****mM) were added after 3****min. Glutamate release was monitored by measuring the increase of fluorescence (excitation and emission wavelengths of 340 and 460****nm, respectively) resulting from NADPH being produced by the oxidative deamination of released glutamate by GDH. In the absence of axonal connectivity, synaptosome is not amenable to electrical stimulation, so a number of biochemical secretagogues have been developed, including the use of K^+^ channel blockers like 4-aminopyridine (4-AP), high external [K^+^] or direct mediation of Ca^+^ entry using Ca^2+^ ionophores such as ionomycin [24]. 4-AP destabilizes the membrane potential and is thought to cause repetitive spontaneous Na^+^ channel-dependent depolarization that closely approximates *in vivo* depolarization of the synaptic terminal, leading to the activation of voltage-dependent Ca^2+^ channels (VDCCs) and neurotransmitter release [25]. Elevated extracellular KCl depolarizes the plasma membrane by shifting the K^+^ equilibrium potential above the threshold potential for activation of VDCCS, which leads to Ca^2+^ entry and neurotransmitter release, while Na^+^ channels are inactivated [26]. Comparison of the effects of berberine under 4-AP and KCl stimulation protocols therefore makes it possible to distinguish between modulatory pathway that target: 1) ionic channels involved in maintaining the plasma membrane potential vs. 2) the VDCCs coupled to glutamate release directly [27].

4-AP (1****mM), high external KCl (15****mM), or ionomycin (5 µM) was added after 10****min of incubation to stimulate glutamate release. Data were accumulated at 2****s intervals. A standard of exogenous glutamate (5****nmol) was added at the end of each experiment and the fluorescence change produced by the standard addition was used to calculate the released glutamate as nanomoles glutamate per milligram synaptosomal protein (nmol/mg). Release values quoted in the text and expressed in bar graphs represent levels of glutamate cumulatively release after 5****min of depolarization and are indicated as nmol/mg/5****min. Cumulative data were analyzed using Lotus 1-2-3.

### Plasma membrane potential

The plasma membrane potential was determined with a membrane-potential-sensitive dye, DiSC_3_(5) [28]. Synaptosomes were resuspended in HBM and incubated in a stirred and thermostatted cuvette at 37 °C in a Perkin-Elmer LS-55 spectrofluorimeter. After 3****min incubation, 5 µM DiSC_3_(5) was added to the synaptosomes and allowed to equilibrate before the addition of CaCl_2_ (1.2****mM) after 4****min incubation. Then, 4-AP (1****mM) was added to depolarize the synaptosomes at 10****min, and DiSC_3_(5) fluorescence was determined at excitation and emission wave lengths of 646****nm and 674****nm, respectively. Cumulative data were analyzed using Lotus 1-2-3 and expressed in fluorescence units.

### Cytosolic free Ca^2+^concentration ([Ca^2+^]_C_)

The [Ca^2+^]_C_ was assayed by on-line fluorimetry as described previously [22]. Synaptosomes (0.5****mg/ml) were resuspended in 1****ml of HBM containing 0.1****mM CaCl_2_ and loaded with 5 µM fura-2-acetoxymethyl ester (Fura-2-AM) for 30****min at 37 °C. Synaptosomes were washed with HBM by centrifugation, resuspended in 2****ml of HBM with BSA, and placed in a Perkin-Elmer LS-55 spectrofluorometer at 37 °C with stirring in the presence of 1.2****mM CaCl_2_. Synaptosomes were incubated for 10****min in the presence of berberine (10 µM) prior to depolarization with 4-AP (1****mM). Fura-2-Ca fluorescence was determined at excitation wavelengths of 340 and 380****nm (emission wavelength, 505****nm) and data accumulated at 2 s intervals. [Ca^2+^]_C_ (nM) was calculated by using calibration procedures [29] and equations described previously [30]. Cumulative data were analyzed using Lotus 1-2-3.

### Western blotting

Synaptosomes from control and drug-treated groups were lysed in Tris–HCl buffer solution, pH 7.5, that contained 20****mM Tris–HCl, 1% Triton, 1****mM EDTA, 1****mM EGTA, 150****mM NaCl, 2.5****mM sodium pyrophosphate, 1****mM β-glycerophosphate, 1****mM phenylmethanesulfonyl fluoride, 1****mM sodium orthovanadate, and 1 µg/ml leupeptin. Lysates were clarified by centrifugation, and protein concentration was determined by the Bradford method. Equal amounts of proteins were subjected to SDS-PAGE (7.5%) and transferred onto nitrocellulose membranes. Membranes were washed with Tris-buffered saline (TBS) that contained 5% low-fat milk and incubated with appropriate primary antibodies (anti-phospho-ERK1/2, 1∶2000; anti-ERK1/2, 1∶1000; anti-phospho-synapsin-I (Ser^62^/Ser^67^), 1∶1000; anti-synapsin I, 1∶500; β-actin, 1∶500) at 4°C for overnight. After three washes with TBS, membranes were incubated for 1****h at room temperature with the secondary horseradish peroxidase-conjugated antibody (1∶3000). The membranes were then washed at least three times with TBS and visualized using the enhanced chemiluminescence system (Amersham, Buckinghamshire, UK). The level of phosphorylation was assessed by band density, which was quantified by densitometry. Densitometric quantification of bands were analyzed using Syngene software (Synoptics, Cambridge, UK).

### Data Analysis

Data are expressed as mean ± SEM. The data reported were analyzed by the unpaired Student's *t* test or by one-way ANOVA with LSD comparisons *post hoc* tests for multiple comparisons. Analysis was completed via software SPSS (17.0; SPSS Inc., Chicago, IL). P<0.05 was considered to represent a significant difference.

## Results

### Berberine inhibits 4-AP-evoked glutamate release from rat cerebrocortical synaptosomes

**Figure 1:**
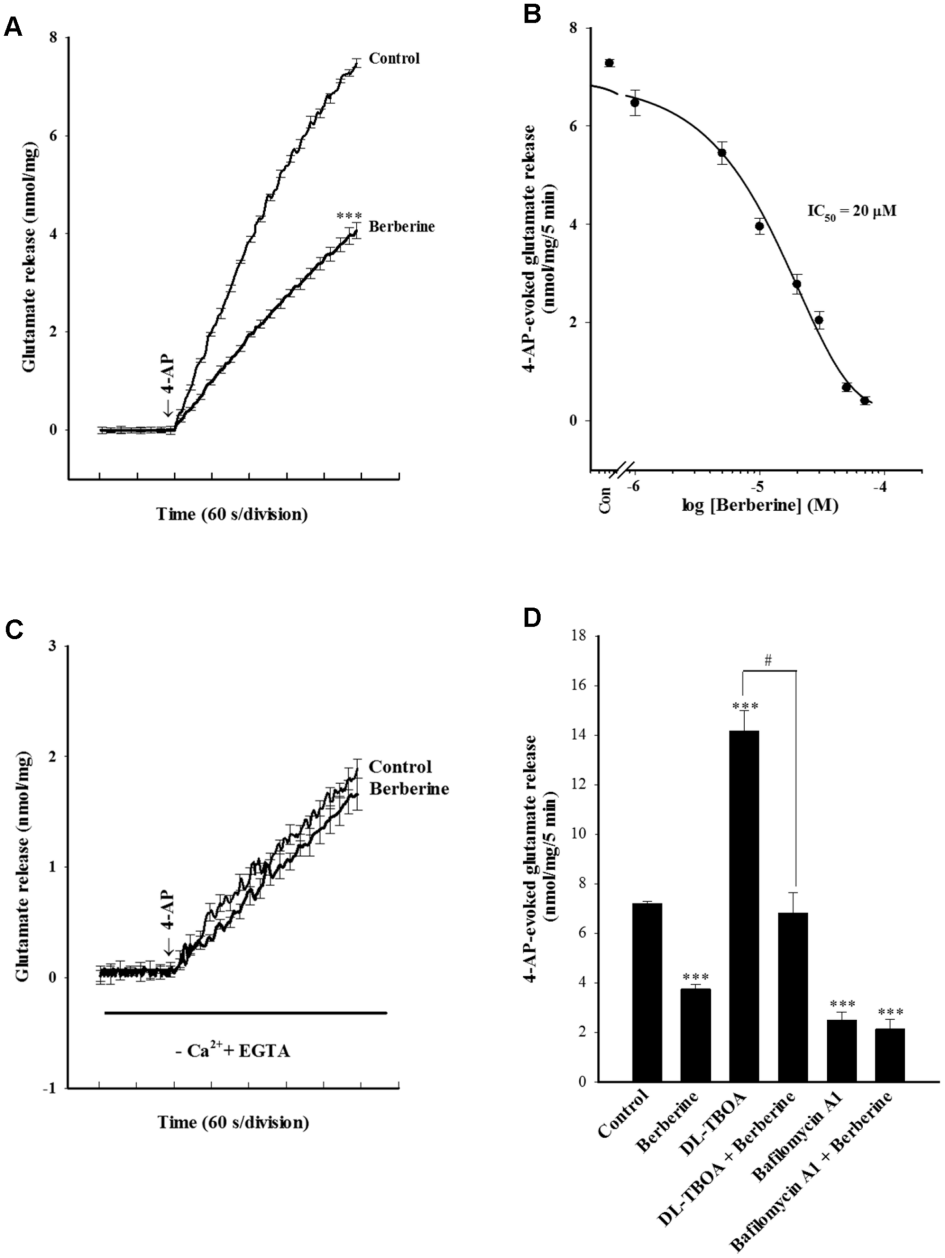
Berberine inhibits 4-AP-induced glutamate release from rat cerebrocortical nerve terminals; this effect is due to a decrease in vesicular exocytosis. Glutamate release (+Ca^2+^; A) and Ca^2+^-independent glutamate release (-Ca^2+^; C) was measured under control conditions or in the presence of 10 µM berberine added 10 min prior to the addition of 4-AP. Ca^2+^-independent release was assayed by omitting CaCl_2_ and adding 300 µM EGTA 10 min prior to depolarization. B: Dose-response curves of decrease in 4-AP-evoked glutamate release in the presence of berberine. D: Glutamate release was evoked by 1 mM 4-AP in the absence and presence of 10 µM berberine and absence and presence of 10 µM DL-TBOA, or 0.1 µM bafilomycin A1. DL-TBOA or bafilomycin A1 was added 20 min before depolarization, while berberine was added 10 min before depolarization. Results are mean ± SEM of 5–6 independent experiments. ***, P<0.001 versus control group; #, P<0.05 versus DL-TBOA-treated group.

4-AP (1****mM) evoked glutamate release of 7.3±0.1****nmol/mg/5****min from cerebral cortex synaptosomes in the presence of 1.2****mM CaCl_2_ (Figure 1A). Preincubation with berberine (10 µM) before 4-AP addition inhibited glutamate release to 3.9±0.2****nmol/mg/5****min (P<0.001), without altering the basal release of glutamate (Figure 1A). The IC_50_ value for berberine inhibition of 4-AP-evoked glutamate release, derived from a log dose-response curve (Figure 1B), was 20 µM. Given the robust repression of evoked glutamate release seen with 10 µM berberine, this concentration was used in subsequent experiments to evaluate the mechanisms that underlie the ability of berberine to reduce glutamate release.

We next investigated whether the inhibition of 4-AP-evoked glutamate release by berberine was mediated by an effect on exocytotic vesicular release, or on Ca^2+^-independent release attributable to cytosolic efflux via reversal of the glutamate transporter [31]. First, we examined the effect of berberine on the Ca^2+^-independent glutamate release. The Ca^2+^-independent glutamate release was measured by depolarizing the synaptosomes with 1****mM 4-AP in extracellular-Ca^2+^-free solution that contained 300 µM EGTA. Under those conditions, the release of glutamate was 1.8±0.1****nmol/mg/5****min. This Ca^2+^-independent glutamate release evoked by 4-AP was, however, not affected by 10 µM berberine (1.7±0.2****nmol/mg/5****min; Figure 1C). Next, we used DL-TBOA, a non-selective inhibitor of all excitatory amino acid transporter (EAAT) subtypes, to examine the effect of berberine on 4-AP-evoked glutamate release. In the presence of DL-TBOA, although 4-AP-evoked glutamate release was increased by the inhibitor (because of inhibition of reuptake of released glutamate) (P<0.001), berberine (10 µM) still significantly reduced the 4-AP-induced release of glutamate (P<0.05; Figure 1D). Third, the effect of berberine on 4-AP-evoked glutamate release was examined in the presence of bafilomycin A1, which causes the depletion of glutamate in synaptic vesicles. In contrast to DL-TBOA, bafilomycin A1 (0.1 µM) reduced control 4-AP (1****mM)-evoked glutamate release (P<0.001), and prevented the inhibitory effect of berberine (10 µM) on 4-AP-evoked glutamate release (Figure 1D). These results suggest that the berberine-mediated inhibition of 4-AP-evoked glutamate release is mediated by a reduction in the Ca^2+^-dependent exocytotic component of glutamate release.

### Berberine does not alter the synaptosomal membrane potential but reduces depolarization- induced increase in [Ca^2+^]_C_

**Figure 2:**
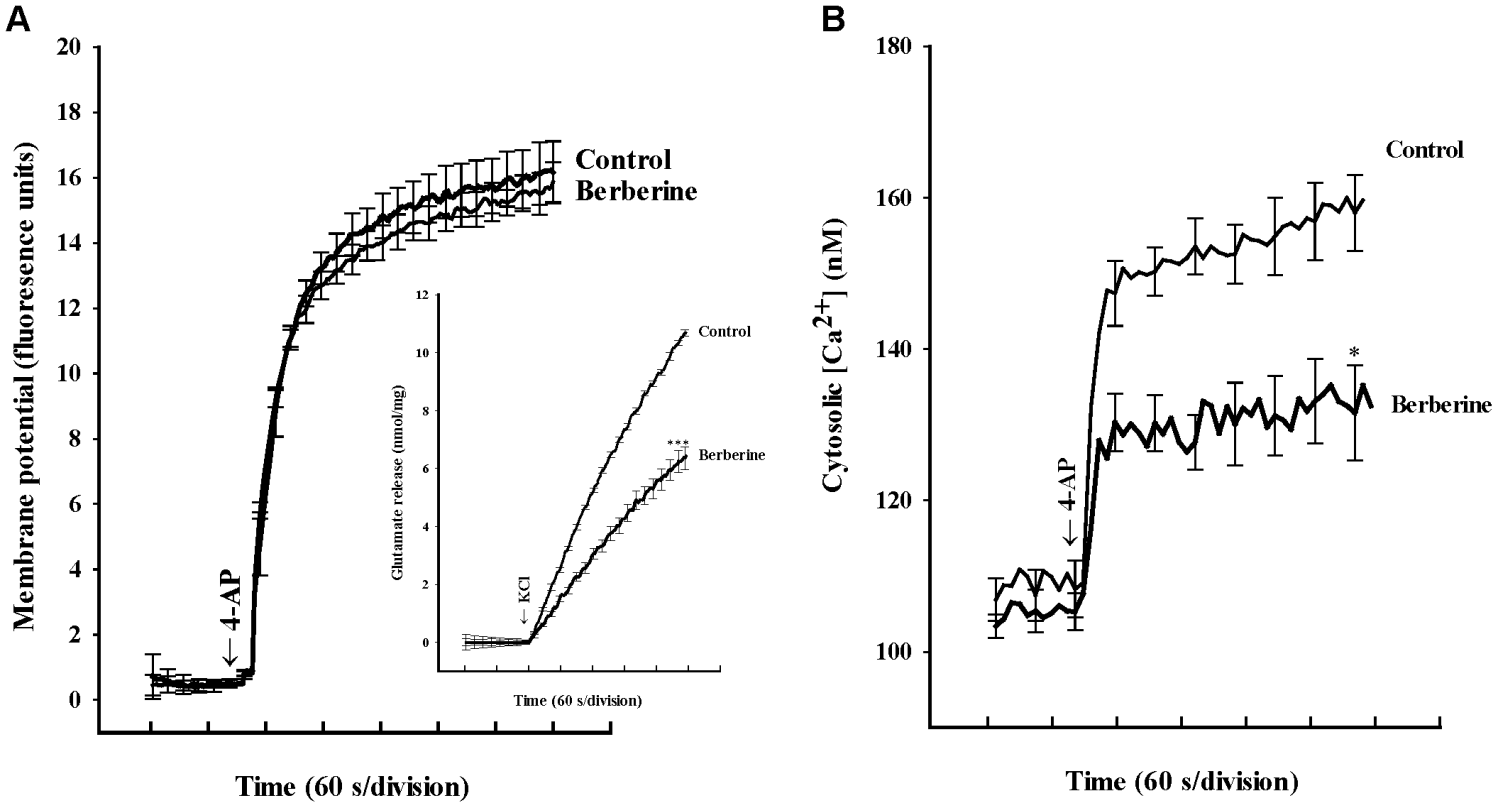
Berberine does not alter the synaptosomal membrane potential but reduces the 4-AP-induced increase in cytosolic Ca^2+^ concentration ([Ca^2+^]_C_). A: Synaptosomal membrane potential monitored with DiSC_3_(5) in the absence (control) and in the presence of 10 µM berberine, added 10 min before depolarization with 1 mM 4-AP. Inset: Glutamate release was induced by 15 mM KCl in the absence (control) or presence of 10 µM berberine, added 10 min before depolarization. B: Cytosolic free Ca^2+^ concentration (nM) was monitored using Fura-2 in the absence (control) and in the presence of 10 µM berberine, added 10 min before depolarization with 1 mM 4-AP. Results are mean ± SEM of 5 independent experiment. ***, P<0.001,* *, P<0.01 versus control group.

To further understand the mechanism responsible for the berberine-mediated inhibition of glutamate release, we used a membrane-potential-sensitive dye, DiSC_3_(5), to determine the effect of berberine on the synaptosomal plasma membrane potential under resting conditions and on depolarization. As shown in Figure 2A, 4-AP (1****mM) caused an increase in DiSC_3_(5) fluorescence. Application of berberine (10 µM) for 10****min before 4-AP addition did not alter the resting membrane potential, and produced no significant change in the 4-AP-mediated increase in DiSC_3_(5) fluorescence. This result indicates that the observed inhibition of evoked glutamate release by berberine is unlikely to have been caused by a hyperpolarizing effect of synaptosomal plasma membrane potential, or attenuation of the depolarization produced by 4-AP. Confirmation that the berberine effect did not impinge on synaptosomal excitability was obtained with experiments using a alternative secretagogue, high external KCl. 15****mM KCl evoked controlled glutamate release of 10.7±0.1****nmol/mg/5****min, which was reduced to 6.4±0.4****nmol/mg/5****min in the presence of 10 µM berberine (P<0.001; Figure 2A, inset).

To monitor berberine-dependent changes in intraterminal Ca^2+^ levels directly, we carried out on-line fluorescent assays using the Ca^2+^ indicator Fura-2. As shown in Figure 2B, 4-AP (1****mM) evoked a rise in [Ca^2+^]_C_, from 108.3±3.7****nM to a plateau level of 159.7±5.2****nM. Application of berberine (10 µM) did not affect basal Ca^2+^ levels, but caused a ∼17% decrease in the 4-AP-evoked rise in [Ca^2+^]c (133.1±6.1****nM; P<0.01; Figure 2B).

### Berberine reduces a Cav2.1 (P/Q-type) channel-coupled glutamate release component

**Figure 3:**
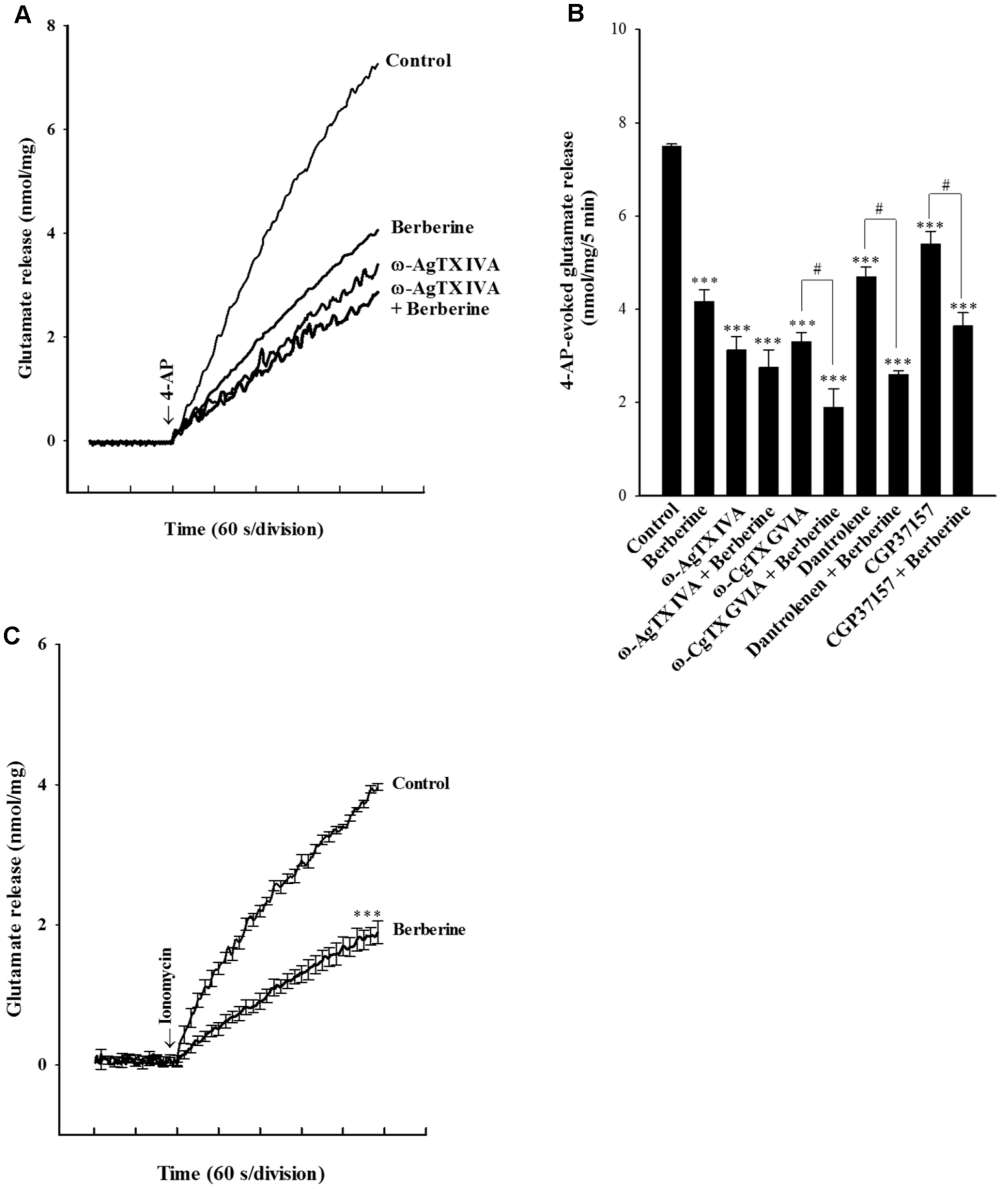
Blockade of Ca_v_2.1 channels eliminates the inhibitory effect of berberine on 4-AP-induced glutamate release. A: Glutamate release was evoked by 1****mM 4-AP in the absence (control) or presence of 10 µM berberine, 0.5 µM ω-AgTX IVA, 0.5 µM ω-AgTX IVA and 10 µM berberine. B: Quantitative comparison of the extent of glutamate release by 1****mM 4-AP in the absence or presence of 10 µM berberine, and absence and presence of 0.5 µM ω-AgTX IVA, 2 µM ω-CgTX GVIA, 100 µM dantrolene, or 100 µM CGP37157. C: Glutamate release was evoked by 5 µM ionomycin in the absence (control) or presence of 10 µM berberine added 10****min depolarization. Berberine was added 10****min before depolarization, whereas the other drugs were added 30****min before depolarization. Results are mean ± SEM of 4–7 independent experiments. ***, P<0.001 versus control group; #, P<0.05 versus ω-CgTX GVIA-, dantrolene-, or CGP37157-treated group.

In the adult rat cerebrocortical nerve terminal preparation, the release of glutamate evoked by depolarization relies is supported by Ca_v_2.2 (N-type) and Ca_v_2.1 (P/Q-type) channels [32], [33]. In order to determine whether the decrease in Ca^2+^ channel activity was involved in the berberine-mediated inhibition of 4-AP-evoked glutamate release, we examined the effects of the Ca^2+^ channel antagonist ω-conotoxin GVIA (ω-CgTX GVIA) and ω-agatoxin IVA (ω-AgTX IVA), which selectively block Ca_v_2.2 and Ca_v_2.1 channels, respectively [34], [35]. In Figure 3A, glutamate release evoked by 1****mM 4-AP was significantly decreased in the presence of 0.5 µMω-AgTX IVA alone (P<0.001). Although 4-AP-evoked glutamate release was significantly reduced in the presence of berberine (10 µM), this effect was prevented in the presence of ω-AgTX IVA, with the release measured in the presence of ω-AgTX IVA and berberine being similar to that obtained in the presence of ω-AgTX IVA alone (Figure 3A and 3B). In addition, glutamate release evoked by 1****mM 4-AP was significantly decreased in the presence of 2 µM ω-CgTX GVIA alone or 10 µM berberine alone (P<0.001; Figure 3B). However, in the presence of ω-CgTX GVIA (2 µM), 4-AP-evoked glutamate release was further inhibited by berberine (10 µM) (P<0.05; Figure 3B). The additive relationship between ω-CgTX GVIA and berberine indicates that Ca_v_2.2 channels are not involved in the observed inhibition of glutamate release by berberine.

In addition to the Ca^2+^ influx through VDCCs, the release of glutamate evoked by depolarization is reported to be caused by Ca^2+^ release from intracellular stores such as endoplasmic reticulum (ER) and mitochondria [36]. Thus, we examined the effect of dantrolene, an inhibitor of intracellular Ca^2+^ release from endoplasmic reticulum, and 7-chloro-5-(2-chloropheny)-1,5-dihydro-4,1-benzothiazepin-2(3H)-one (CGP37157), a membrane-permeant blocker of mitochondrial Na^+^/Ca^2+^ exchange, on the action of berberine. Figure 3B shows dantrolene (100 µM) reduced control 4-AP-evoked release (P<0.001), indicating that Ca^2+^ release from ER ryanodine receptors contributes significantly to the 4-AP-evoked glutamate release. In the presence of dantrolene, however, berberine (10 µM) still effectively inhibited 4-AP-evoked glutamate release (P<0.05; Figure 3B). Similarly to dantrolene, CGP37157 (100 µM) decreased the release of glutamate evoked by 4-AP (1****mM) (P<0.01), but it had no effect on the berberine-mediated inhibition of 4-AP-evoked glutamate release (Figure 3B).

Although the above-mentioned experiments indicate a correlation of the inhibitory effect of berberine on glutamate release with a suppression of VDCCs, the possibility remains that berberine could inhibit glutamate release by directly affecting the release machinery, downstream of Ca^2+^ influx. To examine this possibility, we examined the effect of berberine on glutamate release induced by ionomycin. The Ca^2+^ ionophore ionomycin causes a direct increase in intrasynaptosomal Ca^2+^ levels without previous depolarization and VDCC activation. Thus, ionomycin-induced release reflects the modulation of release machinery downstream of Ca^2+^ entry [29]. In Figure 3C, the glutamate release triggered by ionomycin (5 µM) was also inhibited by berberine (10 µM) (p<0.001).

### Berberine-mediated inhibition of glutamate release involves a MAPK pathway

**Figure 4:**
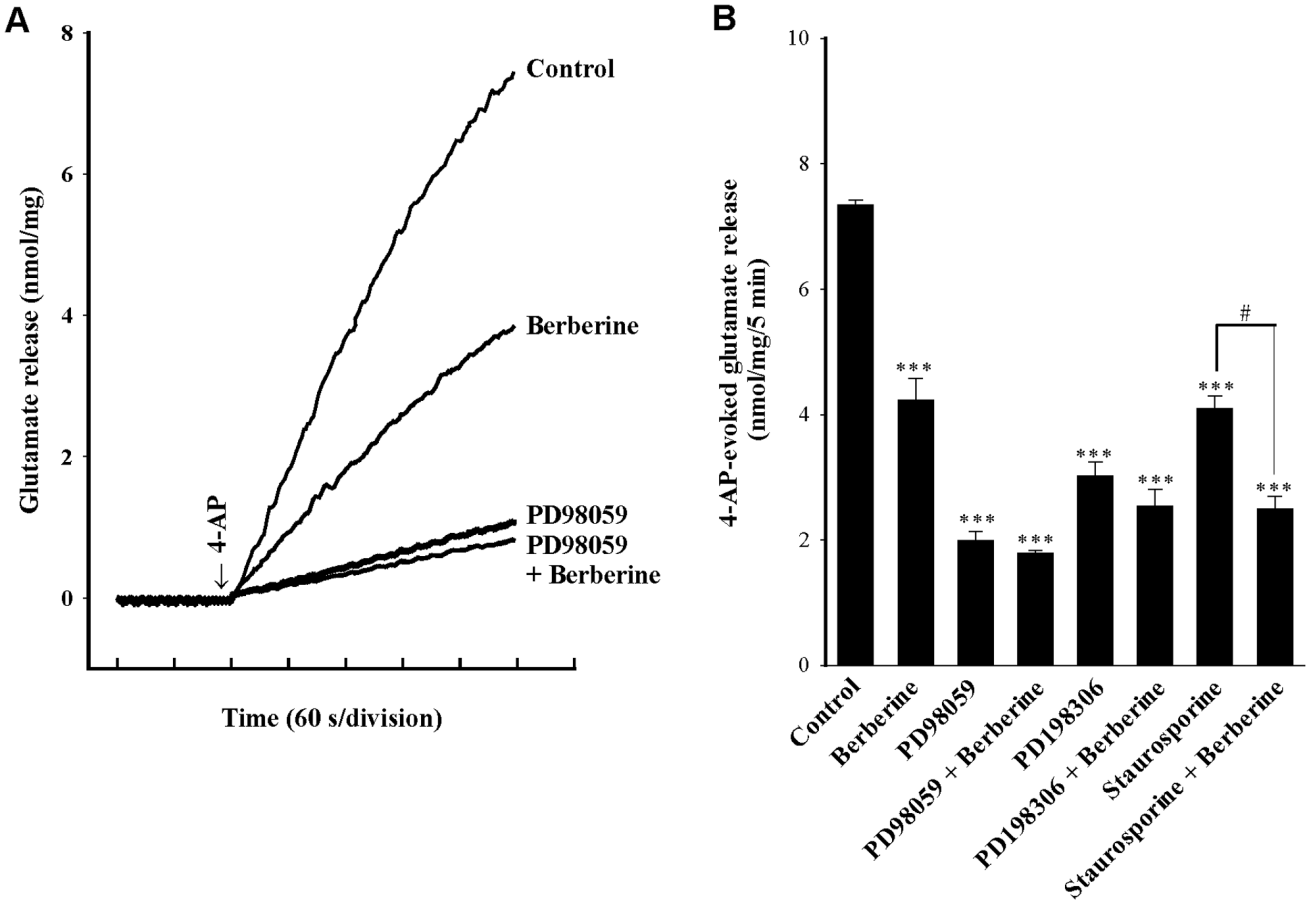
Berberine-mediated inhibition of glutamate release is prevented by the MEK inhibitors PD98059 and PD198306. A: Glutamate release was induced by 1****mM 4-AP in the absence (control) or presence of 10 µM berberine, 50 µM PD98059, or 50 µM PD98059 and 10 µM berberine. B: Quantitative comparison of the extent of glutamate release by 1****mM 4-AP in the absence or presence of 10 µM berberine, and absence and presence of 50 µM PD98059, 100 µM PD198306 or 1 µM staurosporine. PD98059, PD198306, or staurosporine was added 40****min before depolarization, whereas berberine was added 10****min before depolarization. Results are mean ± SEM of 4–5 independent experiments. ***, P<0.001 versus control group; #, P<0.05 versus staurosporine-treated group.

Since the mitogen-activated protein kinase (MAPK) signaling cascade is known to be present at the presynaptic level and has a crucial role in neurotransmitter exocytosis [19], [20], we investigated whether the cascade participated in berberine-mediated inhibition of glutamate release. First, we used PD98059 to prevent the activation of mitogen-activated/extracellular signal-regulated kinase kinase (MEK), the protein kinase upstream of MAPK. Figure 4A shows that control glutamate release evoked by 1****mM 4-AP was reduced by 50 µM PD98059 (P<0.001), reflecting an inhibition of the reported basal MAPK activity present in nerve terminals [20]. Although berberine (10 µM) reduced the 4-AP-evoked glutamate release (P<0.001), this effect was prevented by the pretreatment with PD98059, with the release measured in the presence of PD98059 and berberine being similar to that obtained in the presence of PD98059 alone (Figure 4A and 4B). Similar results were observed with the another MEK inhibitor PD198306 (100 µM; Figure 4B). In contrast, staurosporine, at concentrations 1 µM that inhibit PKC and PKA activities, reduced control 4-AP (1****mM)-evoked glutamate release (P<0.001), but it failed to influence the ability of berberine to inhibit 4-AP-evoked release of glutamate (Figure 4B).

### Berberine decreases the phosphorylation of ERK1/2 and synapsin I

**Figure 5:**
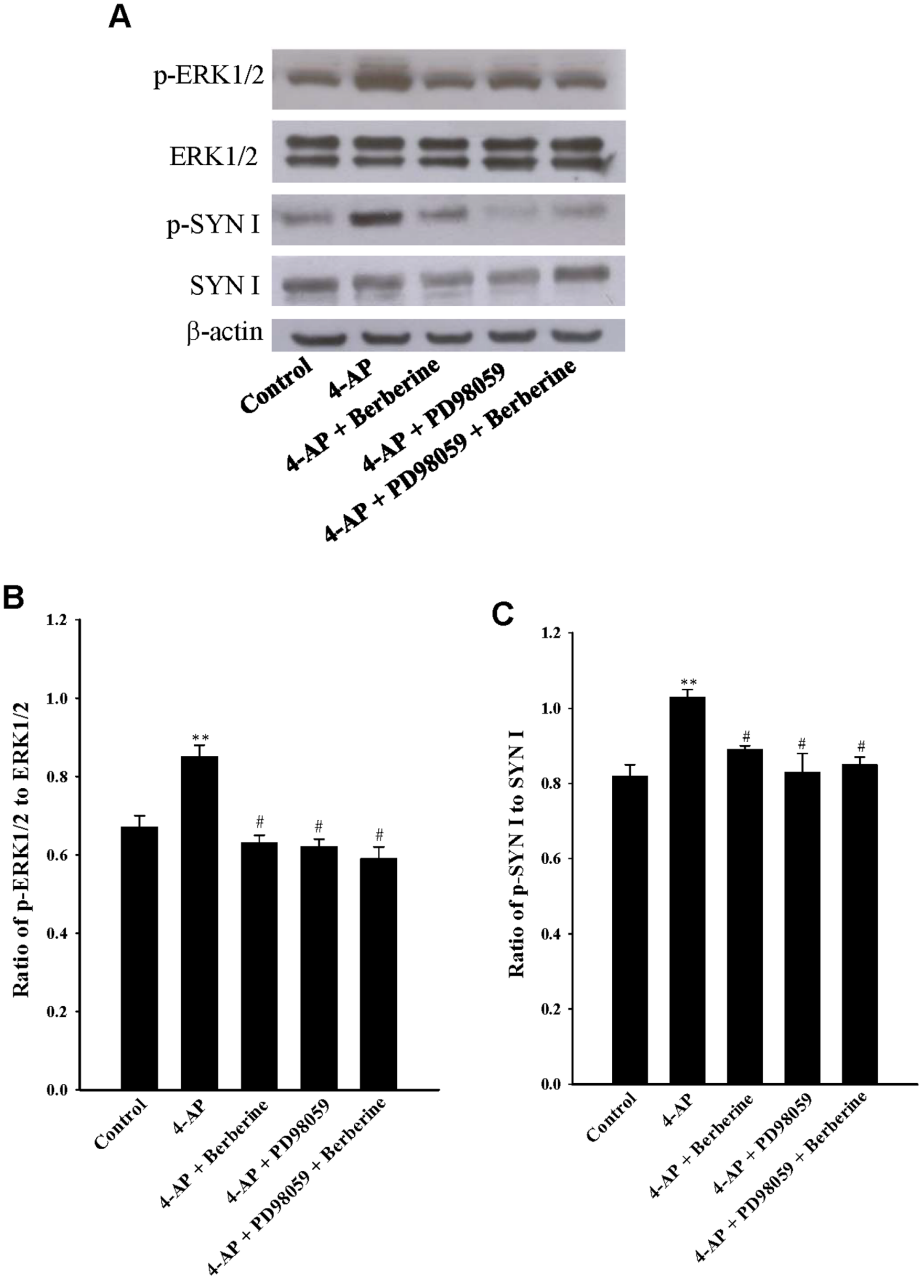
Berberine decreases 4-AP-induced phosphorylation of ERK1/2 and synapsin I, and this effect is prevented by PD98059. A: The representative photographs show levels of p-ERK1/2, ERK1/2, p-SYN I, SYN I, and β-actin in the absence (control) or presence of 1****mM 4-AP, 1****mM 4-AP+10 µM berberine, 1****mM 4-AP+50 µM PD98059, or 1****mM 4-AP+50 µM PD98059+10 µM berberine. β-actin was used as a loading control. PD98059 was added 40****min before 4-AP addition, whereas berberine was added 10****min before depolarization. Quantitative analysis of the ratio of p-ERK1/2 to ERK1/2 (B) or p-SYN I to SYN I (C) was performed. Results are mean ± SEM of 4–6 independent experiments. **, P<0.01 versus control group; #, P<0.05 versus 4-AP-treated group.

To confirm that the MAPK signaling pathway was suppressed by berberine during its inhibition of 4-AP-evoked glutamate release, we determined the effect of berberine on the phosphorylation of extracellular signal-regulated kinase 1 and 2 (ERK1/2) in cerebrocortical synaptosomes. Figure 5 shows that depolarization of synaptosomes with 1****mM 4-AP in the presence of 1.2****mM CaCl_2_ increased the phosphorylation of ERK1/2 (P<0.01). When synaptosomes were pretreated with berberine (10 µM) or PD98059 (50 µM) for 10****min before depolarization with 4-AP, a significant decrease in the 4-AP-induced ERK1/2 phosphorylation was observed (P<0.05). Furthermore, the action of berberine on the 4-AP-induced ERK1/2 phosphorylation was prevented in the presence of PD98059 (Figure 5A and 5B). Similar results were obtained from analysis of phosphorylation of synaptic vesicle-associated protein synapsin I (SYN I), which is the major presynaptic substrate for MAPK/ERK (Jovanovic et al., 2000) (Figure 5A and 5C).

### Berberine-mediated inhibition of evoked glutamate release is prevented in synapsin I-deficient mice

**Figure 6:**
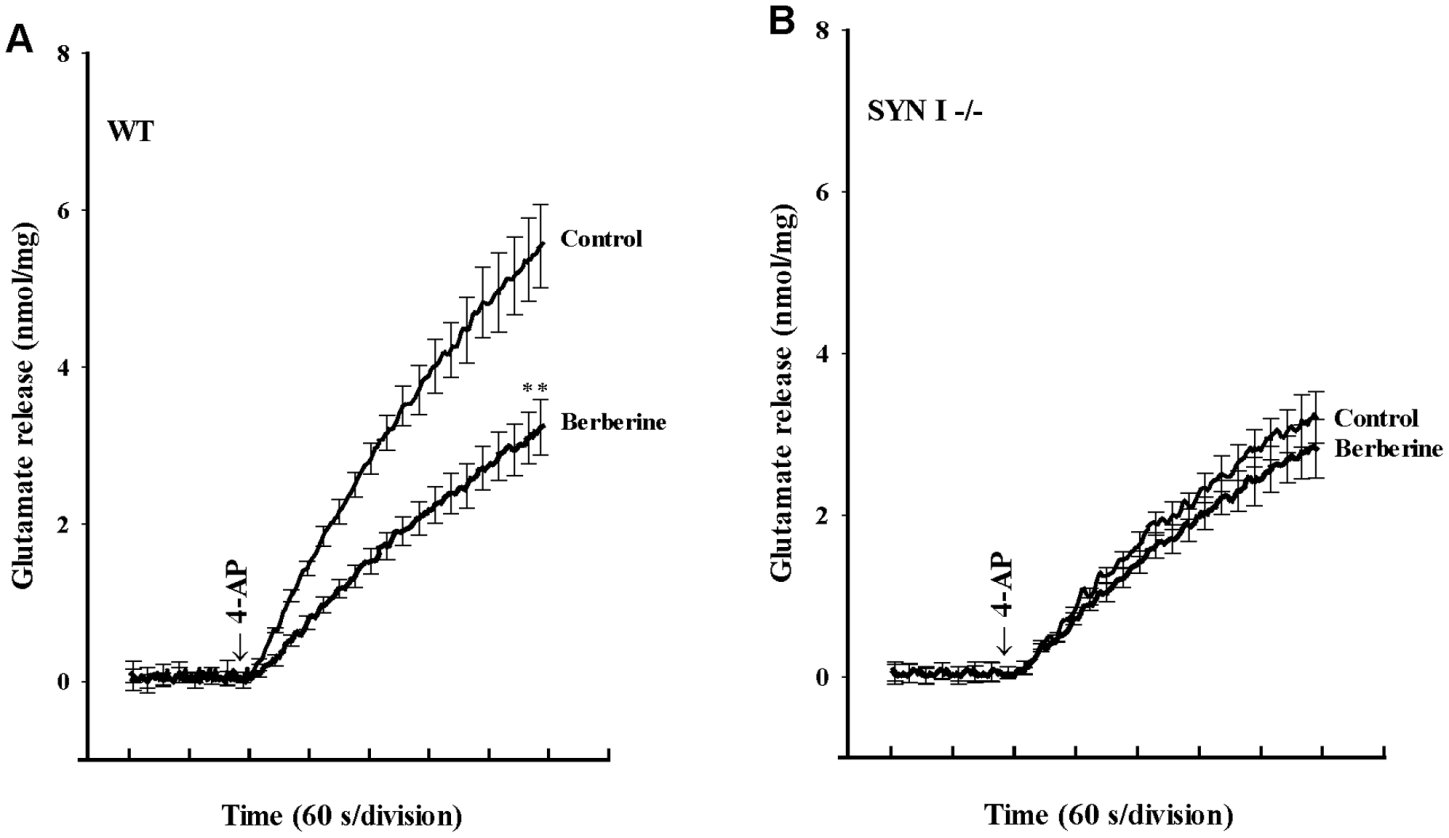
Berberine-mediated inhibition of evoked glutamate release is prevented in synaptosomes from synapsin I-deficient mice. Synaptosomes from wild-type (WT; A) and synapsin I-deficient (SYN I−/−; B) mice were preincubated for 10 min under standard conditions in the absence (control) or presence of berberine (10 µM) followed by the addition of 1 mM 4-AP stimulation. Results are the mean ± SEM values of 5 independent experiments. **, P<0.01 versus control group.

To further evaluate the possibility that the synapsin I is important in the observed inhibition of glutamate release by berberine, we carried out experiments utilizing synaptosomes prepared from wild-type and synapsin I-deficient mice. In wild-type mice, glutamate release evoked by 4-AP (1****mM) under control conditions (5.6±0.5****nmol/mg/5****min) was significantly reduced by berberine (10 µM, 3.2±0.4****nmol/mg/5****min; P<0.01; Figure 6A). Glutamate release evoked by 4-AP (1****mM) was also reduced in synapsin I-deficient mice (3.1±0.3****nmol/mg/5****min; P<0.01; Figure 6B). However, the inhibition of release by berberine observed in wild-type synaptosomes was prevented in synaptosomes isolated from synapsin I-deficient mice (2.8±0.4****nmol/mg/5****min; Figure 6B).

## Discussion

To the best of our knowledge, this study represents the first examination of the effect of berberine on endogenous glutamate release at the presynaptic level. We have used a synaptosomal model to assess the characteristics and mechanism of inhibition of 4-AP-evoked glutamate release by berberine in the cerebral cortex.

The glutamate release induced by 4-AP is known to have 2 components. The first is a physiologically relevant Ca^2+^-dependent component, which is produced by exocytosis of glutamate-containing synaptic vesicles. The second is a Ca^2+^-independent component that results from prolonged depolarization that causes a membrane-potential-mediated shift of the glutamate transporter steady-state toward the outward direction, to affect cytosolic glutamate efflux [27], [31]. In the present study, we found that berberine failed to inhibit the release of glutamate evoked by 4-AP in the absence of extracellular Ca^2+^ (Ca^2+^-independent release). Furthermore, the inhibitory effect of berberine on 4-AP-evoked glutamate release was effectively prevented by bafilomycin A1, which depletes the glutamate content of synaptic vesicles, but not by DL-TBOA, a non-selective inhibitor of all EAAT subtypes. These results suggest that berberine-mediated inhibition of glutamate release impinges on the Ca^2+^-dependent component of release rather than the Ca^2+^-independent efflux due to reversal of the glutamate transporter.

In synaptic terminals, activation of Na^+^ channels or inhibition of K^+^ channels is known to regulate membrane excitability and consequently the amount of transmitter release [37], [38], [39]. Inhibition of Ca^2+^-dependent glutamate release by berberine could be ascribed to an alteration of the synaptosomal plasma membrane potential and downstream modulation of Ca^2+^ influx into the terminal, or a direct regulation of VDCCs affecting Ca^2+^ entry. The first possibility is unlikely for two reasons. First, 4-AP- versus KCl-evoked glutamate release are significantly inhibited by berberine. Both of these depolarizing treatments are thought to activate VDCCs coupled to glutamate release similarly, and therefore, should reflect this by qualitatively similar modulation, if this occurs at the level of the VDCC. Where the two depolarizing paradigms differ is that, whereas 4-AP-evoked glutamate release involves the action of Na^+^ and Ca^2+^ channels, 15****mM external KCl-evoked glutamate release only involves Ca^2+^ channels [25], [26]. Thus, the data presented indicate that Na^+^ channels are not involved in the berberine-mediated inhibition of glutamate release. Second, 4-AP-mediated depolarization, probed using the membrane-potential-sensitive dye DiSC_3_(5), is unaffected by berberine (indicating a lack of effect on K^+^ conductance). These results suggest that decrease in evoked glutamate release seen with berberine is not due to a reduction of synaptosomal excitability resulting from ion channel (e.g., Na^+^ or K^+^ channels) modulation. Our finding disagrees with previous electrophysiological data showing that berberine inhibits K^+^ currents in acutely isolated rat hippocampal CA1 pyramidal neurons [40]. The reason for this discrepancy between the current and previous study is unclear, but may be related to the different experimental models applied.

If it is not the modulation of synaptosomal excitability, then the locus of action of berberine must lie further downstream in the stimulus-exocytosis coupling cascade. Using Fura-2, we have demonstrated that berberine reduces 4-AP-evoked increase in [Ca^2+^]_C_. In synaptic terminals, a depolarization-induced increase in [Ca^2+^]_C_, coupled to glutamate release, is mediated by extracellular Ca^2+^ influx through Ca_v_2.2 and Ca_v_2.1 channels and Ca^2+^ release from intracellular stores such as endoplasmic reticulum and mitochondria [32], [33], [36]. In the present study, although blockade of Ca_v_2.2 channels (by ω-CgTX GVIA) reduced 4-AP-evoked glutamate release, the residual release was still inhibited by berberine. In contrast, Cav2.1 channel block by ω-AgTX IVA prevented the inhibitory effect of berberine on 4-AP-evoked glutamate release, suggesting the involvement of Ca_v_2.1channels. On the other hand, the inhibition of Ca^2+^ release from ER and mitochondria could be excluded. This is because neither dantrolene, an inhibitor of intracellular Ca^2+^ release from the endoplasmic reticulum ryanodine receptors, nor CGP37157, a mitochondrial Na^+^/Ca^2+^ exchange blocker, affected the inhibitory effect of berberine on 4-AP-evoked glutamate release. Although there is no direct evidence that berberine acts on presynaptic Ca^2+^ channels, these data point to the suppression of presynaptic Ca_v_2.1 channels as the mechanism underlying the inhibition of glutamate release by berberine. However, it remains unclear how berberine modulates the Ca_v_2.1channels. Thus, a direct interaction between berberine and presynaptic Ca_v_2.1 channels should be considered when determining the possible mechanism of berberine-mediated presynaptic inhibition. In addition, the present study directly determined whether berberine might affect glutamate release through a mechanism independent of the Ca^2+^ entry using a Ca^2+^ ionophore to stimulate release. Berberine inhibited ionomycin-induced glutamate release without any modulatory effects on ion channels, suggesting, therefore, that at least one locus of berberine action lies downstream of the Ca^2+^ entry. Collectively, these data suggest that berberine contributes to the decrease in glutamate release, not only by attenuating voltage-dependent Ca^2+^ entry, but also by directly interfering with the exocytotic machinery release itself.

A role for the MAPK/ERK pathway in the berberine-mediated inhibition of glutamate release is suggested in this study, based on the following results: (1) the inhibitory effect of berberine on 4-AP-evoked glutamate release was prevented by the MEK (MAP kinase kinase) inhibitors PD98059 and PD198306; (2) the PKC and PKA inhibitor staurosporine did not have any effect on berberine action, which demonstrated some specificity for berberine action on the MAPK pathway; (3) berberine decreased the 4-AP-induced phosphorylation of ERK1/2 and synapsin I at MAPK-specific sites 4 and 5, this phenomenon was also prevented by the MEK inhibitor PD98059; and (4) the inhibition of glutamate release by berberine was strongly attenuated in mice lacking synapsin I. It has been demonstrated that depolarization-stimulated Ca^2+^ entry leads to MAPK/ERK activation and to phosphorylation of synapsin I at sites 4 and 5. This phosphorylation reaction promotes dissociation of synaptic vesicles from the actin cytoskeleton, thereby making more vesicles available at the active zone for neurotransmitter exocytosis, resulting in increased glutamate release [41], [42], [43], [44]. Accordingly, our results imply that the suppression of MAPK/ERK-dependent synapsin I phosphorylation and the consequent decreased availability of synaptic vesicles is involved in the observed berberine-mediated inhibition of glutamate release. However, aside from synapsin I, the possible involvement of other synaptic proteins should be considered. Synapsin II and synapsin III, for example, are reported to be phosphorylated by MAPK [45].

Berberine has been confirmed to penetrate the blood-brain barrier [46] and possess neuroprotective activity both in animal and cell culture models of neurotoxicity such as ischaemia and Alzheimer's disease [5], [6], [7], [8], [47]. The exact mechanism responsible for the neuroprotective effect of berberine remains to be further clarified. Previous research had already demonstrated that this beneficial effect is associated with antioxidant action, scavenging oxygen-free radicals, reducing intracellular calcium concentration, inhibiting N-methyl-D-aspartate (NMDA) receptor activity, and anti-inflammation [2], [3]. In this work, reducing glutamate release from nerve terminals may explain, in part, the neuroprotective mechanism of berberine. For instance, an excessive release of glutamate is widely considered to be one of the molecular mechanisms of neuronal damage in several neurological states, including ischemic brain damage and neurodegenerative diseases [12], [13]. Furthermore, some neuroprotective agents have been revealed to decrease glutamate release in human and rat brain tissues [48], [49], [50]. On the other hand, berberine-mediated inhibition of glutamate release in our study is dose dependent, maximal at 50 µM, with a IC_50_ of 20 µM. Berberine, at 5–25 µM, has been shown to attenuate glucose-deprivation- or NMDA-induced neurotoxicity [51]. Furthermore, administering berberine (20–50****mg/kg) to animals protects against ischemia-induced brain damage, and improves β-amyloid-induced memory impairment [5], [8]. The present study is consistent with these reports, but the neural protective effects of berberine were also observed in a lower concentration range, i.e., 10****nM-2 µM [5], [6].

In conclusion, this is the first study to demonstrate that berberine inhibits glutamate release from cerebrocortical synaptosomes by a reduction of Ca^2+^ influx through Ca_v_2.1 channels, in the absence of any effect on nerve terminal excitability. Furthermore, this release inhibition is likely to depend, at least in part, on the suppression of MAPK/ERK/synapsin I pathway. The relevance of our finding to *in vivo* clinical situations remains to be determined. However, this finding may provide further understanding of the mode of berberine action in the brain, thereby emphasizing the therapeutic potential of this compound in the treatment of a wide range of neurological and neurodegenerative disorders.
